# Assessing midbrain neuromelanin and its relationship to reward learning in anorexia nervosa: Stage 1 of a registered report

**DOI:** 10.1002/brb3.3573

**Published:** 2024-06-19

**Authors:** Stuart B. Murray, Joel P. Diaz‐Fong, Vienna W. T. Mak, Jamie D. Feusner

**Affiliations:** ^1^ Department of Psychiatry & Behavioral Sciences University of Southern California Los Angeles California USA; ^2^ Centre for Addiction and Mental Health Toronto Ontario Canada; ^3^ Institute of Medical Science, Temerty Faculty of Medicine University of Toronto Toronto Ontario Canada; ^4^ Semel Institute for Neuroscience & Human Behavior, Department of Psychiatry & Biobehavioral Science University of California, Los Angeles Los Angeles California USA; ^5^ Department of Psychiatry, Temerty Faculty of Medicine University of Toronto Toronto Ontario Canada; ^6^ Department of Women's and Children's Health Karolinska Institutet Stockholm Sweden

**Keywords:** anorexia nervosa, appetitive conditioning, neuromelanin, reward

## Abstract

**Introduction:**

Anorexia nervosa (AN) is a debilitating and potentially chronic eating disorder, characterized by low hedonic drive toward food, which has been linked with perturbations in both reward processing and dopaminergic activity. Neuromelanin‐sensitive magnetic resonance imaging (MRI) is an emerging method to index midbrain neuromelanin—a by‐product of dopaminergic synthesis. The assessment of midbrain neuromelanin, and its association with AN psychopathology and reward‐related processes, may provide critical insights into reward circuit function in AN.

**Methods:**

This study will incorporate neuromelanin‐sensitive MRI into an existing study of appetitive conditioning in those with AN. Specifically, those with acute and underweight AN (*N* = 30), those with weight‐restored AN (*N* = 30), and age‐matched healthy controls (*N* = 30) will undergo clinical assessment of current and previous psychopathology, in addition to structural neuromelanin‐sensitive MRI, diffusion MRI, and functional MRI (fMRI) during appetitive conditioning.

**Conclusion:**

This study will be among the first to interrogate midbrain neuromelanin in AN—a disorder characterized by altered dopaminergic activity. Results will help establish whether abnormalities in the midbrain synthesis of dopamine are evident in those with AN and are associated with symptomatic behavior and reduced ability to experience pleasure and reward.

## Graphical abstract

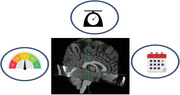

## 1 INTRODUCTION

Anorexia nervosa (AN) is a debilitating and potentially life‐threatening eating disorder that yields mortality rates among the highest of any psychiatric illness, with a crude mortality rate of 5.6% (Arcelus et al., 2011). While the benefits of specialized treatments remain limited (Murray et al., 2019), AN may run a chronic and relapsing illness course, with more than half of those afflicted with AN still meeting diagnostic criteria more than two decades after illness onset (Fichter et al., 2017). Moreover, AN has been associated with multisystemic organ damage, bone disease, and structural and functional brain impairment (Mitchell & Crow, 2006), which cumulatively render functional impairments comparable to those seen in schizophrenia (Vos & Mathers, 2000) and autism (Whiteford et al., 2015). Upon the backdrop of risk associated with AN, and the modest rates of treatment response, the need to elucidate the brain‐based mechanisms of AN is critical so that precision treatments can be developed (Schmidt & Campbell, 2013).

Symptomatically, AN is characterized by self‐imposed starvation, emaciation, an intense fear of weight gain, and a marked disturbance in the perception of one's body shape and weight (American Psychiatric Association, 2013). Behaviorally, this typically manifests as an avoidance of palatable foods, despite their near universal hedonic properties in individuals without AN (Kringelbach, 2004). In exploring the mechanisms of this pervasive food restriction, a growing body of evidence has indicated aberrant experiences of reward among those with AN (Cowdrey et al., 2013; Kaye et al., 2013). For instance, among those with AN, (i) olfactory, visual, and taste cues are reliably rated as *less* pleasant than observed in healthy controls (HCs) (Cowdrey et al., 2013; Jiang et al., 2010); (ii) *negative* affect is reliably elevated after meals (Anderson et al., 2014); and (iii) food consumption is reported to be *aversive* rather than hedonic (Bruch, 1978; Bruch et al., 1994). However, altered reward responding extends beyond food‐specific contexts, suggesting potentially phenotypic perturbations in reward‐related processes. To that end, diminished hedonia and a reduced drive to pursue novelty and fun (Boehm et al., 2018; Harrison et al., 2014) are evident, alongside an unease in social and sexual relationships (Gonidakis et al., 2015; Pinheiro et al., 2010), which persists even after remission.

At the neurobiological level, structural abnormalities in the brain's reward circuitry have been observed in AN, which is evident in both gray matter structures and white matter fiber tracts. Reduced gray matter volume is evident in key nodes of the brain's reward circuit, including the ventral striatum and orbitofrontal cortex (OFC) (Frank et al., 2013; Titova et al., 2013), and aberrant connectivity of white matter tracts in reward systems has also been observed (Cha et al., 2016; Via et al., 2014). In white matter tracts connecting key reward circuit regions of the ventral tegmental area (VTA) and the nucleus accumbens (NAc), we previously found in adolescents with AN abnormal microstructure in the form of reduced neurite and myelin density and reduced orientation dispersion (Murray et al., 2023). Further, orientation dispersion in this tract—a measure of tract “fanning,” which, in general, is negatively correlated with fractional anisotropy (FA) (Zhang et al., 2012)—was associated with subjective reward responsiveness.

With regard to functional activity in the brain's reward circuit, mixed results have emerged from functional magnetic resonance imaging (fMRI) studies of reward‐related tasks to date. In the context of food cues, fMRI studies have generally noted diminished corticostriatal activity in AN (Brooks et al., 2011; Jiang et al., 2019), which extends to the taste, smell, and sight of palatable foods (Holsen et al., 2012; Jiang et al., 2019; McFadden et al., 2014; Oberndorfer et al., 2013). However, other studies suggest *hyperactivity* in reward‐related regions such as the ventral striatum (Cowdrey et al., 2011) and amygdala (Vocks et al., 2010) in response to palatable food cues, despite those with AN implicitly and explicitly stating “liking” and “wanting” these foods less than controls (Cowdrey et al., 2013). In concert, heightened activity in prefrontal regions including the medial prefrontal cortex and dorsolateral prefrontal cortex following exposure to palatable food cues has driven several hypotheses suggesting elevated top‐down inhibition of reward processing (Kerr et al., 2017; Uher et al., 2003; Uher et al., 2004).

Furthermore, studies assessing dopaminergic function—a critical neurotransmitter relating to reward learning and hedonic responding—suggest trait‐level abnormalities in those with AN. For instance, *reduced* concentrations of CSF homovanillic acid—a dopamine metabolite—have been found in those with AN, even after recovery (Kaye et al., 1999), and greater dopamine receptor availability has been noted in the ventral striatum of those with AN (Frank et al., 2005). In keeping with these data, aberrant neural response to hedonic cues extends beyond food cues. For instance, those with AN show abnormal corticostriatal activity in response to monetary rewards (Wagner et al., 2007), which accords with behavioral data illustrating a diminished predilection toward valuing and pursuing immediate monetary gain (Steinglass et al., 2017).

Paradoxically, those with AN describe dietary restriction and the hunger state as intensely rewarding (Kaye et al., 2013, 2009), raising the intriguing question of whether dimensions of reward processing are intact but have antonymic associations to those in healthy individuals. Relatedly, empirical data have demonstrated elevated ventral striatum activity following exposure to images of thin people (Fladung et al., 2010, 2013), and elevated OFC activity has been observed in response to low‐calorie foods, which is associated with fasting levels of acylated ghrelin (Holsen et al., 2014). These data may suggest that hunger could amplify the reward processing of low‐calorie foods.

In the context of these mixed multimodal results, the question of whether there exists a fundamental aberrancy in hedonic processes in AN remains of critical importance. However, several key gaps remain in our understanding of reward processes in AN. First, the capacity for de novo learning of hedonic associations in AN has been largely understudied. Ascertaining the integrity of appetitive learning systems in AN, for which an ongoing indifference to palatable food consumption often persists despite specialized treatments (Pike, 1998; Schebendach et al., 2012), is crucial. Moreover, the central goal of treatment across *all* treatment modalities is oriented toward enhancing the incentive salience of food cues, promoting approach behaviors, and facilitating greater food consumption. As such, elucidating the integrity of appetitive learning in AN, and its decay and reinstatement, will inform efforts to optimize patient outcomes by targeting the incentive salience of recovery‐congruent cues and food‐related approach behaviors.

To address this gap, our group recently developed an fMRI study protocol to leverage an appetitive Pavlovian conditioning paradigm to probe hedonic learning systems in AN (Murray et al., 2022). Specifically, we will interrogate the acquisition, extinction, spontaneous reinstatement, and recovery of associative reward learning to a disorder‐neutral and positively valenced cue. While appetitive conditioning studies frequently employ palatable food cues (i.e., chocolate, sucrose) as the unconditioned stimulus (US), the use of palatable tastants in AN population is problematic as it is conflated with the existing *aversive* associations to palatable food tastants. Disorder‐neutral, positively valenced USs are therefore warranted.

However, in further understanding the functionality of reward‐related processed in AN, the dopaminergic system is a potentially critical factor, considering both its potent role in reward learning processes (Keiflin & Janak, 2015; Wise, 2004) and its sensitivity to weight loss and food restriction (Frank et al., 2019). For instance, animal studies have illustrated that food restriction and weight loss drive a sensitization of brain reward pathways (Avena et al., 2008; Carr, 2007; Carr et al., 2003) and dopaminergic neuronal activity (Oinio et al., 2017). This adaptive sensitization of D1 and D2/D3 receptors in response to food restriction is thought to drive greater motivation to forage and seek food (Frank et al., 2019). In keeping, the dopamine‐mediated prediction error response is elevated in the NAc, head of the caudate, and insula in underweight adolescents with AN, although this appears to normalize upon weight restoration (DeGuzman et al., 2017).

Thus far, the methods used to interrogate the dopaminergic system in AN have been limited to positron emission tomography (PET) and single‐photon emission computed tomography (SPECT) studies. One novel method of indexing dopaminergic activity, which may be less acutely sensitive to food restriction and weight loss, is neuromelanin‐sensitive magnetic resonance imaging (NM‐MRI). Neuromelanin, which is a byproduct of dopamine and catecholamine synthesis, is found in dopaminergic neurons in the substantia nigra and in the noradrenergic neurons of the locus coeruleus (Graham, 1979). The oxidation of cytosolic dopamine, advanced by ferric iron, reacts with cytosolic proteins and is polymerized, which yields an undegradable iron–melanin–protein complex (Graham, 1979). Following macroautophagy and fusion with lysosomes, these neuromelanin organelles accumulate over the lifespan and are not subject to proteasome degradation (Graham, 1979). As such, neuromelanin is likely not acutely affected by acute weight loss and food restriction—which therefore offers a novel window into assessing the cumulative activity of the dopaminergic system in midbrain regions. Of relevance to AN, single‐unit recordings in response to both (i) food and (ii) conditional stimuli signaling food delivery have been observed in dopaminergic neurons in the substantia nigra (Ljungberg et al., 1992; Mirenowicz & Schultz, 1994, 1996), underscoring the role of nigrostriatal dopaminergic pathways in food‐related reward processing.

NM‐MRI leverages the highly paramagnetic nature of iron–neuromelanin complexes, utilizing reduced magnetization transfer and shortened T1 relaxation times to generate high signal intensities in brain regions containing neuromelanin (Sulzer et al., 2018). Evidence from a series of validation studies suggests that NM‐MRI is a reliable proxy for dopaminergic activity in the striatum (Cassidy et al., 2019). A meta‐analysis indicated that NM‐MRI may reliably delineate those with Parkinson's disease—a disorder characterized by abnormalities in dopaminergic processes—from those without and with 89% sensitivity and 83% specificity (Cho et al., 2021). Further, a meta‐analysis of studies in schizophrenia found elevated neuromelanin contrast‐to‐noise ratio (NM‐CNR) in the substantial nigra but not the locus coeruleus (Wieland et al., 2021). However, currently there are no published studies of NM‐MRI in those with AN—a disorder with noted perturbations in dopaminergic processes. Given the cumulative acquisition of neuromelanin in midbrain regions throughout the lifespan, the likelihood of acute changes in neuromelanin in response to starvation and weight loss among those with AN is low. However, whether alterations in dopaminergic synthesis—which can be indexed by NM‐MRI—are related to illness features and reward functioning in AN is an important question. Emerging evidence in broader psychiatry suggests that diminished neuromelanin may be evident in disorders of impulsivity (Cassidy et al., 2019). Moreover, studies of OCD—a disorder highly comorbid with AN—suggest that greater symptom severity and illness duration are independently linked to greater perturbations in midbrain neuromelanin (Pagliaccio et al., 2023). As such, the assessment of midbrain neuromelanin and both illness duration and symptom severity in AN—a disorder highly linked to dopaminergic perturbations and altered hedonic drive—is warranted.

With these considerations, we extended a recently developed multimodal Pavlovian appetitive conditioning study in underweight and weight‐restored AN (WRAN) (Murray et al., 2022), to include the assessment of midbrain neuromelanin in the substantia nigra. Specifically, in addition to indexing the acquisition, extinction, spontaneous reinstatement, and recovery of associative reward learning across multiple units of analyses including subjective experience (self‐report), physiology (heart rate deceleration and pupillary dilation), neural response (brain activity in reward systems), and white matter structural connectivity (from diffusion‐weighted MRI) (Murray et al., 2022), we will also undertake baseline NM‐MRI. In avoiding the use of any cues that may have already acquired a negative valence in those with AN (e.g., food cues), our appetitive conditioning paradigm (Murray et al., 2022) will employ positively valenced, socially rewarding yet symptom‐neutral infant laughter sounds at the US, which adolescents with AN typically rate positively (Murray et al., 2022). The study aims to assess (i) group differences in NM‐CNR in the substantia nigra pars compacta (SNpc) among underweight restricting‐type AN (AN‐R) patients, weight‐restored AN‐R (WRAN‐R) patients, and age‐matched HCs, (ii) assess the extent to which NM‐CNR in the SNpc accords with illness duration among those with AN, (iii) assess the extent to which NM‐CNR in the SNpc accords with blood oxygen level dependent (BOLD) activity in nodes of the reward circuit during the acquisition of positively valenced associations, (iv) assess the extent to which NM‐CNR in the SNpc accords with structural connectivity in the reward circuit, and (v) assess the relationship between NM‐CNR in the SNpc and self‐reported AN trait appetitive motivation. With empirical findings noting stable sex differences in associative learning, the proposed study will include only female participants.

We hypothesize that (1) compared to controls, those with both acute AN and WRAN will demonstrate reduced NM‐CNR in the SNpc; (2) NM‐CNR in the SNpc will be associated with illness duration in both the underweight (e.g., current illness duration to date) and WRAN (e.g., overall illness duration) groups, respectively; (3) there will be an association between NM‐CNR in the SNpc and subjective appetitive motivation on the behavioral activation scale (BAS) across participants; and (4) there will be an association between NM‐CNR in the SNpc and structural connectivity (as indexed by FA) between VTA/SNpc and the NAc across participants. Lastly, (5) we will undertake exploratory assessments of the concordance between NM‐CNR in the SNpc and BOLD response in the NAc and SNpc during the acquisition stage of our conditioning paradigm, without a priori hypotheses.

## 2 MATERIALS AND METHODS

### 2.1 Reproducibility

This study has been registered with ClinicalTrials.gov (NCT05531604), and upon completion of data collection, summary data will be made publicly available. In addition, data analytic scripts will be made available upon request to the corresponding author.

### 2.2 Participants

In keeping with our parent study^47^, participants will be female adolescents and young adults (aged 12–22 years), who are (1) acute and underweight AN (*N* = 30), (2) WRAN (*N* = 30), and (3) age‐matched HCs (*N* = 30). All participants will undergo structural NM‐MRI, diffusion MRI, and fMRI during appetitive conditioning. Additional recruitment and assessment information, such as inclusion and exclusion criteria, are described in detail in Murray et al. (2022).

### 2.3 Neuromelanin‐sensitive MRI

NM‐MRI will be acquired with a GE Discovery 3T scanner with a 32‐channel head coil. We will acquire two‐dimensional gradient response echo sequence with magnetization transfer contrast (2D GRE‐MT) (Plane = Oblique; repetition time [TR] = 284 ms; echo time [TE] = 4.1 ms; flip angle = 50°; in‐plane resolution = 0.43 × 0.43 mm^2^; partial brain coverage with field of view [FOV] = 512 × 512 mm; number of slices = 10; slice thickness = 2.5 mm; slice gap = 0 mm; MT offset = 1200 Hz; acquisition time = 08:01 min:s). The image slab will be oriented along the anterior commissure–posterior commissure line, with the top slice approximately 3 mm above the floor of the third ventricle to provide coverage of portions of the midbrain containing the SNpc. Three‐dimensional T1‐weighted images (MPRAGE; 0.8 mm^3^) using HCP Lifespan protocols (humanconnectome.org) will be acquired and used for registration. Both NM‐MRI and T1‐weighted images will be visually inspected for artifacts. Preprocessing of the NM‐MRI will be performed using steps that have previously been shown to have high test–retest reliability (Ljungberg et al., 1992). Specifically, preprocessing will be performed using the Advanced Normalization Tools (ANTs; http://stnava.github.io/ANTs/), which will consist of (1) brain extraction of the T1‐weighted image, (2) co‐registration of the NM‐MRI to the T1‐weighted image in native space, (3) spatial normalization of the T1‐weighted image to standard MNI space through nonlinear registration, (4) application of the estimated transformation (determined in step 3) to the NM‐MR image, and lastly (5) spatial smoothing of the normalized NM‐MRI image using a 1‐mm full‐width half‐maximum (FWHM) 3D Gaussian kernel. For each participant, a mask of the crus cerebri will be created by manual tracing of the NM‐MRI image in MNI space using the Seg3D software (www.seg3d.org).

### 2.4 Diffusion MRI

Diffusion‐weighted images will be acquired with a single‐shot spin‐echo echo planar sequence (TR = 4100 ms, TE = 81.7 ms; flip angle = 90°; FOV = 140 × 140 mm; resolution = 1.7 mm^3^; *b* = 3000, 2000, 1000, and 500 seconds/mm^2^; number of slices = 81; number of directions = 102; acquisition time = 07:11 min:s) in the posterior‐to‐anterior direction. A shortened sequence (number of directions = 6) in the anterior‐to‐posterior direction will also be acquired, resulting in pairs of images with reversed phase‐encoding directions (distortions going in opposite directions) that will be utilized for EPI distortion correction. Diffusion MRI preprocessing will be performed using FMRIB's Software Library (FSL; www.fmrib.ox.ac.uk/fsl/). Preprocessing steps will involve using FSL's TOPUP and EDDY tools to estimate and correct the susceptibility‐induced off‐resonance field and correct eddy/current movements. Additional denoising steps such as outlier replacement (Andersson et al., 2017), slice‐to‐volume movement correction (Andersson et al., 2017), and susceptibility‐by‐movement correction (Andersson et al., 2018) will be applied. Bayesian estimation of diffusion parameters (BEDPOSTX) will be used to model and determine the number of crossing fibers per voxel (Jbabdi et al., 2012). Probabilistic tracking with crossing fibers (PROBTRACKX) (Behrens et al., 2007) will be estimated using VTA/SNpc as a seed region and NAc as the target region. Diffusion tensor model will be fitted using DTIFIT to derive FA maps and (exploratory) mean diffusivity (MD) and radial diffusivity (RD) maps.

### 2.5 Outcome measures

#### 2.5.1 Neuromelanin contrast‐to‐noise ratio

NM‐CNR will be calculated as the percent signal difference at each voxel relative to a participant‐specific reference of modal signal intensity in the crus cerebri, a nearby white matter region with minimal neuromelanin content (Cassidy et al., 2019). Average neuromelanin CNR will be extracted for the SNpc based on a probabilistic atlas (Ito et al., 2008).

#### 2.5.2 fMRI BOLD signal

Acquisition and processing of the BOLD response will be performed as described in Murray et al. (2022). To explore the concordance between NM‐CNR in the SNpc and BOLD response in the NAc during the acquisition stage of our conditioning paradigm, probabilistic ROI of the NAc will be obtained using the Reinforcement Learning Atlas (Pauli et al., 2018).

#### 2.5.3 Structural connectivity

The structural connectivity between the VTA/SNpc and the NAc will be determined as average FA (and as exploratory, MD, and RD) values of the right‐ and left‐averaged projection tract connecting the two regions.

### 2.6 Data analysis

#### 2.6.1 Analysis plan

Variable transformations prior to subsequent analyses will be determined based on distribution diagnostics and outlier analysis. To test Hypothesis 1, we will conduct an ANCOVA, controlling for age and pubertal development scale (PDS) scores, to compare mean NM‐CNR in the SNpc among AN, WRAN, and HCs. We will use multiple linear regression to test Hypothesis 2, examining associations between NM‐CNR in the SNpc and illness duration in the AN group, using age and PDS scores as covariates of noninterest. To test Hypothesis 3, we will use linear regression, examining associations between NM‐CNR in the SNpc and behavioral activation scale scores from the BIS/BAS across all participants, using age and PDS scores as covariates of noninterest. To test Hypothesis 4, we will use multiple linear regression to examine associations between NM‐CNR in the SNpc and structural connectivity (FA), controlling for age and PDS scores. For the exploratory analysis, we will use multivariate multiple linear regression to examine associations between NM‐CNR and BOLD signal. Dependent variables will be BOLD signal in the SNpc and the NAc from the CS+ versus CS− contrast (first eigenvariate) during the acquisition phase, and independent variables will be NM‐CNR in the SNpc, group (AN, WRAN, HCs), and group × NM‐CNR interaction, with age and PDS scores as covariates of noninterest.

#### 2.6.2 Power analysis

No published studies to date have assessed NM‐MRI in those with AN for estimates of effect sizes. As this is a secondary data analysis, the sample was powered for the primary outcomes for primary hypotheses of the parent study, as previously outlined (Murray et al., 2022). With a sample size of *n* = 30 AN, *n* = 30 WRAN, and *n* = 30 HCs, for Hypothesis 1, we will have power = .8 at alpha = .05 to detect an effect size of *f* = .33 (medium). For Hypothesis 2 (*n* = 30), we will have power = .8 at alpha = .05 to detect an effect size of *f* ^2 ^= .28 (medium). For Hypotheses 3 and 4 (*N* = 90), we will have power = .8 at alpha = .05 to detect an effect size of *f* ^2 ^= .089 (small).

## 3 CONCLUSION

The study proposed here will systematically assess midbrain neuromelanin in AN, as well as its relationship to appetitive conditioning, and may offer critical insights around dopaminergic system in AN—a disorder that has been centrally linked to perturbed reward‐related mechanisms. However, the vast majority of existing studies assessing dopaminergic functioning in AN to date have been predominantly PET studies. While PET studies can measure pre‐ and postsynaptic dopaminergic functions such as binding of D1 and D2 receptors, dopamine transport binding, and endogenous dopamine synthesis rate (Ito et al., 2008), it is an invasive procedure, involves radiation, and requires more specialized research facilities (Horga et al., 2021). NM‐MRI data are more easily acquired and offer a novel methodology to index the *synthesis* of dopamine, by assessing a direct byproduct of dopamine metabolism in dopaminergic neurons in the substantia nigra (Graham, 1979). In turn, and when taken alongside findings from PET studies, these findings may allow for a more nuanced understanding of the synthesis and uptake of dopamine, respectively, when elucidating dopaminergic abnormalities in AN.

Historically, dopaminergic neurons in the substantia nigra have been thought to be centrally implicated in motor pathways and disorders of movement, such as Parkinson's disease and essential tremor (Biondetti et al., 2021; Isaias et al., 2016). However, single‐unit recordings in response to both (i) food and (ii) conditioned stimuli signaling food delivery have reliably been observed in dopaminergic neurons in the substantia nigra (Ljungberg et al., 1992; Mirenowicz & Schultz, 1994, 1996), underscoring the role of the nigrostriatal dopaminergic pathway in food‐related reward processing.

The current study will investigate these potential connections between reward and brain dopaminergic functioning by measuring neuromelanin in the SNpc. In addition to comparing NM‐CNR among those with AN‐R, those with WRAN‐R, and HCs—to establish if there are abnormal levels in those with AN and who are currently in, or had previously been in, a malnutrition/starvation state—the study will also examine associations with AN illness duration. Further, it will investigate associations between SNpc dopaminergic function and reward behaviors (subjective appetitive motivation) across the sample, as well as the correspondence between NM‐CNR and a blood flow‐related measure of neural activity (BOLD signal) in the SNpc and the NAc (another important reward circuit node) during appetitive conditioning. These additional investigations across the sample promise to contribute to the field's general knowledge of the links between NM‐CNR, as a proxy for dopaminergic synthesis, and both reward behaviors and brain activity in reward systems.

Results of this investigation of dopaminergic synthesis in the substantia nigra among those with AN offer promise in advancing our understanding of the neurobiological underpinnings of AN and may lay the groundwork for developing novel lines of treatment for AN and other psychiatric disorders involving diminished ability to experience pleasure and reward.

## AUTHOR CONTRIBUTIONS

Stuart B. Murray and Jamie D. Feusner led the manuscript development, will coordinate the study, and are responsible for its methodological design. In addition, Stuart B. Murray and Jamie D. Feusner will oversee all diagnostic assessments. Stuart B. Murray, Joel P. Diaz‐Fong, and Jamie D. Feusner developed and pilot tested the appetitive conditioning paradigm. Stuart B. Murray, Joel P. Diaz‐Fong, Vienna W. T. Mak, and Jamie D. Feusner developed the neuroimaging analysis plan. All authors read, edited, and approved the final manuscript.

## CONFLICT OF INTEREST STATEMENT

The authors declare no conflicts of interest.

### PEER REVIEW

The peer review history for this article is available at https://publons.com/publon/10.1002/brb3.3573.

## Data Availability

The datasets generated and analyzed during the current study contain clinical data and are not publicly available due to the protection of participants’ rights to privacy and data protection, but are available from Stuart B. Murray and Jamie D. Feusner upon reasonable request. Summary data will be made available on ClinicalTrials.gov.
